# Teaching Adolescents With Type 1 Diabetes Self-Compassion (TADS) to Reduce Diabetes Distress: Protocol for a Randomized Controlled Trial

**DOI:** 10.2196/53935

**Published:** 2023-12-26

**Authors:** Saunya Dover, Alexandra Ahmet, Karen Bluth, Brian M Feldman, Ellen B Goldbloom, Gary S Goldfield, Sarah Hamilton, Omar Imran, Adam Khalif, Karine Khatchadourian, Sarah Lawrence, Andrew Leonard, Kuan Liu, Yongdong Ouyang, Corien Peeters, Jai Shah, Noah Spector, Caroline Zuijdwijk, Marie-Eve Robinson

**Affiliations:** 1 Children's Hospital of Eastern Ontario Research Institute Ottawa, ON Canada; 2 Division of Endocrinology & Metabolism Children's Hospital of Eastern Ontario Ottawa, ON Canada; 3 Faculty of Medicine University of Ottawa Ottawa, ON Canada; 4 Department of Psychiatry University of North Carolina Chapel Hill, NC United States; 5 Institute of Health Policy, Management and Evaluation Dalla Lana School of Public Health University of Toronto Toronto, ON Canada; 6 Division of Rheumatology Department of Pediatrics The Hospital for Sick Children and University of Toronto Toronto, ON Canada; 7 Harvard Extension School Harvard University Cambridge, MA United States; 8 Clinical Epidemiology Program The Ottawa Hospital Research Institute Ottawa, ON Canada; 9 School of Epidemiology and Public Health University of Ottawa Ottawa, ON Canada; 10 Development & Rehabilitation Children's Hospital of Eastern Ontario Ottawa, ON Canada; 11 Department of Psychiatry McGill University Montreal, QC Canada; 12 Douglas Research Centre Montreal, QC Canada; 13 Eating Disorders Program Children’s Hospital of Eastern Ontario Ottawa, ON Canada

**Keywords:** anxiety, depression, diabetes distress, disordered eating, mental health, mindful self-compassion, pediatrics, randomized controlled trial, type 1 diabetes

## Abstract

**Background:**

Adolescents living with type 1 diabetes (T1D) often experience diabetes distress (DD), a construct distinct from depression or anxiety that refers to the negative emotions that arise from living with and managing diabetes. Self-compassion, which involves being open to one’s own suffering and treating oneself with the same care one would show to loved ones, is associated with better psychological and clinical outcomes among individuals with T1D. Self-compassion is a skill that can be taught and therefore represents an opportunity for intervention.

**Objective:**

The overall aim of this study is to assess the effectiveness of a web-based mindful self-compassion for teens (MSC-T) intervention on improving DD, anxiety, depression, diabetes-related disordered eating, and suicidal ideation experienced by youth with T1D (aged between 12 and 17 years) compared with a waitlist control group (standard of care). We will also explore (1) if the effect of the MSC-T intervention changes over time, (2) if the MSC-T intervention has a positive impact on measures of glycemic control, and (3) if the effect of the MSC-T intervention differs based on self-reported gender.

**Methods:**

We will conduct a single-center, parallel-group randomized controlled trial of 140 adolescents with T1D followed for 12 months. Participants will be randomly allocated (using hidden allocation) in a 1:1 ratio to either the MSC-T intervention or the waitlist control group. Our primary outcome is DD, as measured by the Problem Areas in Diabetes-Teen (PAID-T) version at 3 months. Secondary outcomes, assessed at 3 and 12 months, include anxiety (Generalized Anxiety Disorder 7-item [GAD-7] scale), depression (Patient Health Questionnaire-9 [PHQ-9]), diabetes-related disordered eating (Diabetes Eating Problem Survey-Revised [DEPS-R] version), and suicidal ideation (using 1 question from the PHQ-9).

**Results:**

Study recruitment began in October 2022 and was completed in March 2023, with a total of 141 participants enrolling. Data collection will be ongoing until March 2024. The first results are expected in June 2024.

**Conclusions:**

This study will be the first randomized trial to assess the effectiveness of the web-based MSC-T intervention on adolescents with T1D. Given that adolescence is a period where individuals are typically required to assume more responsibility for their diabetes care, providing adolescents with the tools they need to better manage the stress that often accompanies T1D management is paramount.

**Trial Registration:**

ClinicalTrials.gov NCT05463874; https://clinicaltrials.gov/study/NCT05463874

**International Registered Report Identifier (IRRID):**

DERR1-10.2196/53935

## Introduction

### Background and Rationale

Adolescence constitutes a critical developmental period during which youth establish their autonomy while grappling with the social, emotional, and physical challenges of becoming young adults. This period represents a time of tremendous vulnerability, during which psychiatric and mood disorders often manifest [1]. Adolescence is even more challenging for youth with type 1 diabetes (T1D), as they contend with the daily challenges of disease management in addition to the developmental challenges inherent to this phase of development.

Diabetes distress (DD) is an emotional construct that refers to the negative emotions that arise from living with and managing diabetes. The majority of youth with T1D experience some level of DD [2,3], with adolescent females experiencing greater DD than males [4-6]. More than one-third of youth with T1D suffer from substantial DD [7]. DD is apparent in youth for many reasons, including fear of stigma from peers [8], feeling overwhelmed by their diabetes regimen [9], and worry of hypoglycemia and long-term complications of a potentially fatal condition [9].

Self-compassion, a positive psychological construct, involves being open to one’s own suffering and treating oneself with the same care one would show to loved ones [10]. Youth can have varying degrees of innate self-compassion [11]. Higher levels of self-compassion are associated with lower levels of distress in youth with other chronic medical conditions [12], and it is a psychological characteristic that correlates with favorable outcomes, including psychological well-being, self-care, and hemoglobin A_1c_ (HbA_1c_) among individuals with diabetes [13].

Among adults with diabetes, an intervention to increase self-compassion resulted in reductions in DD [14]. However, this relationship has yet to be shown in youth with T1D. Self-compassion interventions lead to better psychological well-being and decreased psychopathology in healthy adolescents and those with some chronic conditions [15,16]. Since self-compassion is a modifiable behavior on which one can be trained [17,18], we have a largely untapped opportunity for intervention among youth with T1D.

Mindfulness-based therapy has led to short-term improvements in self-reported stress among adults and emerging adults with T1D [19]. However, a sole focus on mindfulness is not sufficient, as it does not provide enough training around active self-comforting in suffering. Mindfulness also does not provide the aptitude to address the feelings of failure and self-blame that are common among youth with T1D [20]. Therefore, our goal is to study the effects of a psychological intervention combining mindfulness and self-compassion (mindful self-compassion for teens [MSC-T]) on DD among youth with T1D.

### Objectives

Our primary objective is to compare DD at 3 months among youth with T1D who participated in the MSC-T intervention to those in a waitlist control group. Our secondary objectives are to (1) compare anxiety, depression, diabetes-related disordered eating, and suicidal ideation at 3 months among youth with T1D who participated in the MSC-T intervention with those in the waitlist control group; and (2) compare DD, anxiety, depression, diabetes-related disordered eating, and suicidal ideation at 12 months among youth with T1D who participated in the MSC-T intervention with those in the waitlist control group. Finally, our exploratory objectives are to (1) explore if the effect of the MSC-T intervention changes over time throughout the 12-month follow-up period, (2) compare glycemic control (HbA_1c_ and time in range) among adolescents with T1D who participated in the MSC-T intervention to those in the waitlist control group, and (3) explore if the effect of the web-based MSC-T intervention on DD differs based on self-reported gender.

### Hypotheses

We hypothesize that adolescents who participate in the MSC-T intervention will report lower DD, anxiety, depression, diabetes-related disordered eating, and suicidal ideation at 3 months compared with the waitlist control group.

## Methods

### Ethical Considerations

This study was reviewed and approved by the local Research Ethics Board at the Children’s Hospital of Eastern Ontario (CHEO; protocol 22/09E, approved July 17, 2022). Following an introduction from a member of the participant’s circle of care, in line with requirements outlined in the Personal Health Information Protection Act, a member of the research team will obtain written informed consent, either in person or remotely, using our REDCap (Research Electronic Data Capture; Vanderbilt University) system [21,22]. Participants will consent on their own behalf and will be compensated with gift cards for completing the study questionnaires (US $15 for baseline, US $20 for week 8, US $30 for month 3, US $30 for month 6, and US $35 for month 12) and up to 15 volunteer hours.

### Study Design

We will conduct a single-center, parallel-group randomized controlled trial assessing the effect of a web-based MSC-T intervention on DD in adolescents with T1D followed for 12 months, compared with a waitlist control group (standard of care).

### Participants and Recruitment

We will recruit 140 adolescents with T1D, aged between 12 and 17 years, from the Diabetes Clinic at CHEO in Ottawa, Ontario, Canada.

### Inclusion and Exclusion Criteria

The inclusion criteria and exclusion criteria for this study are presented in Textbox 1.

**The inclusion and exclusion criteria.**

**Inclusion criteria**

- Aged between 12 and 17 years.
- Diagnosed with type 1 diabetes (T1D) ≥6 months before enrollment.
- Ability to provide informed consent.

**Exclusion criteria**

- Inability or unwillingness to provide informed consent.
- Inability to speak English with enough fluency to complete all study-related tasks.
- Presence of an intellectual disability that would preclude participation in the mindful self-compassion for teens (MSC-T) intervention, as assessed by the treating physician.
- A lifetime diagnosis of a psychotic or bipolar disorder given by a clinician (these often involve intensive treatments with psychological and pharmacologic implications, which may confound our results).
- Presence of acute suicidality at the time of enrollment.
- Active participation in another mental health intervention trial.

### Trial Intervention

**Figure 1:**
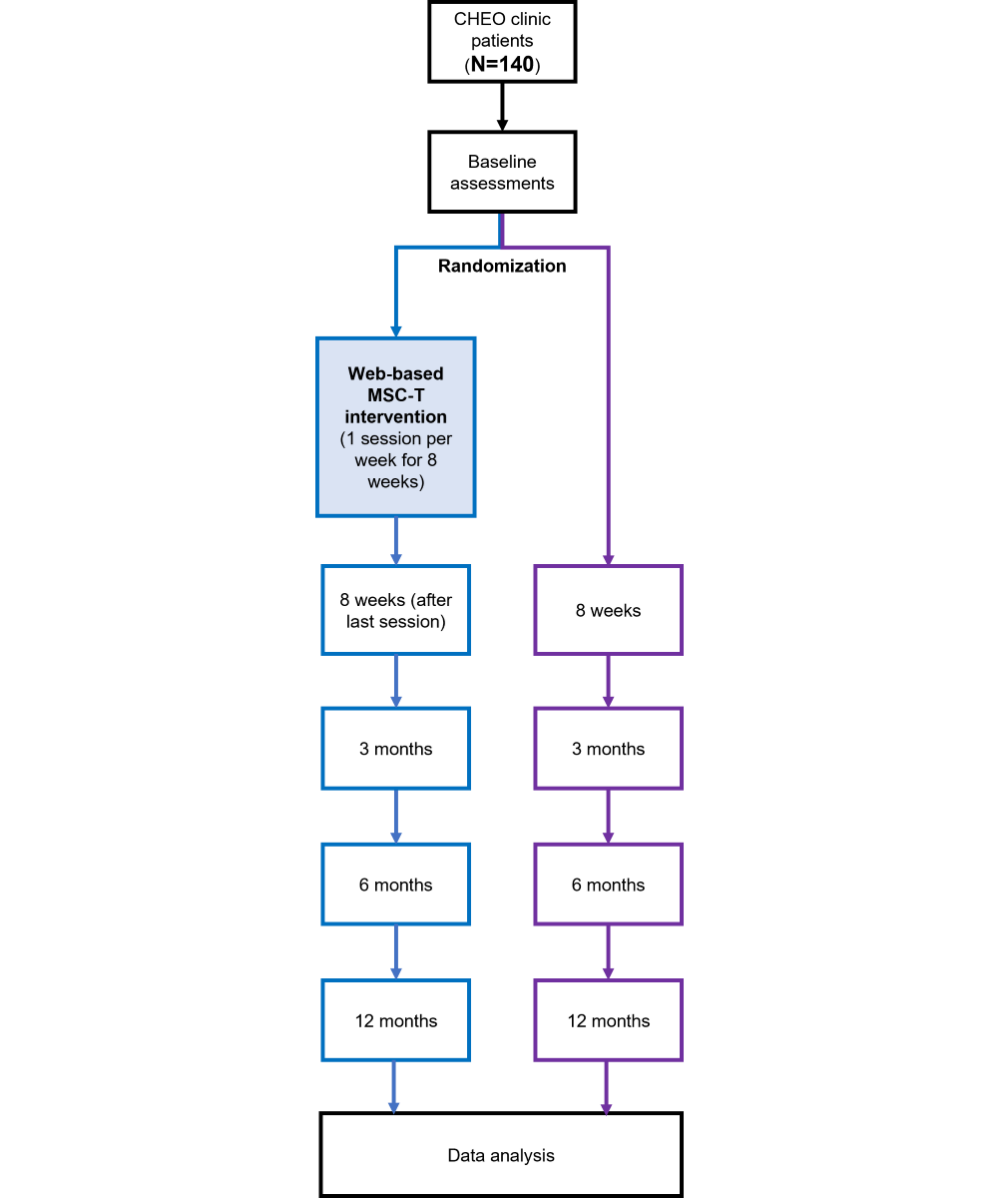
Schematic overview of the study. CHEO: Children’s Hospital of Eastern Ontario; MSC-T: mindful self-compassion for teens.

As shown in Figure 1, participants from the diabetes clinic at CHEO will be randomized to the MSC-T intervention group or the waitlist control group (standard of care).

#### Intervention Group

The MSC-T intervention (Textbox 2) teaches the participants to be open to, rather than disconnected from, their own suffering and treat themselves with the same care they would show to a friend [10]. The program consists of 8 weekly interactive sessions, each lasting 1.5 hours (a total of 12 hours). The program will be led by our collaborators (KB and AL), who are trained, experienced MSC-T instructors. Each session will begin with a brief mindful art activity, which provides a transition to the class. Classes include developmentally appropriate hands-on exercises, videos, games, mindful movement, music meditation, and between-class practice assignments to solidify practices. The session content and structure were reviewed by our people with lived experience collaborators (SH and OI).

MSC-T program fidelity will be determined by (1) assessing the change in self-compassion between baseline and 8 weeks and (2) assessing if the required topics for each MSC-T session were covered and activities performed. Self-compassion will be assessed using the Self-Compassion Scale for Youth, a psychometrically valid and reliable measure of self-compassion for adolescents [23]. To ensure the respective topics are appropriately covered in the MSC-T sessions, a member of the study team will complete a fidelity assessment during each session.

Participants’ satisfaction and acceptability of the intervention will be evaluated using an intervention satisfaction survey, similar to what has been previously used to determine satisfaction with the web-based MSC-T program [18].

**Program summary (sessions and topics) for mindful self-compassion for teens (MSC-T) intervention.**

**Week 1**

Discovering mindful self-compassion: introduction to concepts of mindfulness and self-compassion; safety measures for class established; and both informal and formal practices are introduced.

**Week 2**

Paying attention on purpose: concepts of mindfulness, wandering mind, and default mode network are discussed; mindful eating, “soles of the feet,” and “palm of the hand” meditations are presented.

**Week 3**

Lovingkindness: “lovingkindness” is defined and lovingkindness practice is introduced; participants create their own lovingkindness phrases; adolescent brain development is discussed.

**Week 4**

Self-compassion: this exercise encourages teenagers to turn from the “inner critic” toward the “compassionate voice,” and music meditation is introduced.

**Week 5**

Self-compassion versus self-esteem: the difference between these 2 is elucidated, and the perils of social comparison are discussed.

**Week 6**

- Living deeply: core values exercise; “giving and receiving” meditation is introduced.

**Week 7**

- Managing difficult emotions: “soften, soothe, and allow” practice is introduced; tools to contend with anger and unmet needs are practiced; and 2 developing systems of the adolescent brain are explained.

**Week 8**

- Embracing your life with gratitude: gratitude and self-appreciation practices are presented, and the wrap-up of the course takes place through writing a letter to oneself.

#### Waitlist Control Group

Participants in the wait-list control group will receive standard-of-care treatment as usual. Following the conclusion of the experimental protocol, participants in this group will be given the opportunity to complete the 8-week web-based MSC-T program.

### Randomization Procedures

We will use the randomization module in REDCap [21,22] to allocate participants to their group. The randomization table will be created by a study team member who is not directly involved in patient recruitment or data collection using R (R Core Team) [24]. The trial coordinator will generate the allocation for each participant following obtaining informed consent and completion of the baseline assessments (ie, there will be hidden allocation).

Participants will be randomly allocated to either the MSC-T intervention group or the waitlist control group using a 1:1 ratio block randomization. We will stratify each block by age group (12-14 years [early adolescence] or 15-17 years [middle adolescence]). The cohort will be divided by these age groups to deliver the intervention with the goal of fostering a developmentally appropriate and open environment. Stratification by age group is therefore necessary to ensure an equal age distribution between the treatment groups, since the developmental age of a participant could impact their response to the intervention [25]. We will also randomly vary the block size, with blocks of either 6 or 8, to prevent prediction of group allocation as participants are accrued [26].

### Data and Outcome Measures

We will collect the following demographic information at baseline: age, postal code (to determine socioeconomic status using neighborhood-level material and social deprivation indices [27,28]), optional self-reporting of gender identity (participants will be presented with a range of options [29,30]; Table 1), race or ethnicity, and indigeneity [31] (Table 2), and participant’s social context (living arrangements, etc). Medical history will be collected at baseline, and at intervals, it will be reviewed at each follow-up visit to determine if participants have been newly diagnosed with a serious mental illness (exclusion or withdrawal criteria).

**Table 1:** Gender reporting options to be presented to participants. The presented gender reporting options are in line with accepted standards for determining gender identity in research [29,30]. Participants will be informed that the provision of this information is voluntary and that they will be allowed to select more than one.

Self-reported option	Analysis group
Male	Male
Female	Female
Transgender	Will depend on if another option is selected
Transgender boy or transgender male	Male
Transgender girl or transgender female	Female
Genderqueer	Gender diverse
Gender expansive	Gender diverse
Androgynous	Gender diverse
Nonbinary	Gender diverse
Two-spirited	Gender diverse
Third gender	Gender diverse
Agender	Gender diverse
Not sure or questioning	Gender diverse
Other	Will depend on free text
Prefer not to say	Will be excluded from gender analysis

**Table 2:** Ethnicity and Indigenous identity questions and responses to be presented to participants. The categories are in line with accepted standards for reporting ethnicity from the Canadian Institute for Health Information [31]. Participants will be informed that the provision of this information is voluntary and that they will be allowed to select more than one. Participants will be asked to answer the following question: “In our society, people are often described by their race or racial background. These are not based in science, but our race may influence the way we are treated by individuals and institutions, and this may affect our health and health outcomes related to diabetes. Which category or categories best describe you? Check all that apply.”

Response category	Examples
Black	African, African Canadian, and Afro-Caribbean descent
East Asian	Chinese, Japanese, Korean, and Taiwanese descent
Indigenous (First Nations, Inuk/Inuit, Métis)	First Nations, Inuk or Inuit, and Métis descent
Latin American	Hispanic or Latin American descent
Middle Eastern	Arab, Persian, and West Asian descent (eg, Afghan, Egyptian, Iranian, Kurdish, Lebanese, and Turkish)
South Asian	South Asian descent (eg, Bangladeshi, Indian, Indo-Caribbean, Pakistani, and Sri Lankan)
Southeast Asian	Cambodian, Filipino, Indonesian, Thai, Vietnamese, or other Southeast Asian descent
White	European descent
Other category	Includes options not described above
Do not know	N/A^a^
Prefer not to answer	N/A

^a^N/A: not applicable.

Our primary outcome, DD, has been shown to be the psychological construct most related to metabolic outcomes in adolescents with T1D [3]. DD will be measured using the Problem Areas in Diabetes-Teens (PAID-T) version [32], a validated measure to evaluate diabetes-specific emotional distress in adolescents with high internal consistency (Cronbach α=.96) [32]. It consists of 26 items, each scored on a 6-point ordinal scale, allowing for the calculation of an overall distress score of 26-156, with scores of <70, 70-90, and >90 representing none-to-mild, moderate, and severe DD, respectively [33]. The PAID-T has excellent psychometric properties [34], is sensitive to change from behavioral intervention [35], and is a commonly used measure to assess DD in adolescents [7,36].

Our secondary outcomes are anxiety, depression, diabetes-related disordered eating, and suicidal ideation. Symptoms of anxiety will be measured using the Generalized Anxiety Disorder 7-item (GAD-7) scale, a self-report tool to detect clinically significant anxiety symptoms. The GAD-7 is scored from 0-21, with 0-4, 5-9, 10-14, and 15-21 indicating minimal, mild, moderate, and severe anxiety symptoms, respectively [37]. It has been validated in adolescents [38] and has previously been used to assess adolescents with T1D [39,40].

Symptoms of depression will be measured using the Patient Health Questionnaire-9 (PHQ-9) by self-report [41]. The PHQ-9 is a valid and reliable screening tool for identifying adolescents meeting the Diagnostic and Statistical Manual of Mental Disorders, Fourth Edition (DSM-IV) criteria for major depression [41]. Scores range from 0-27, and in adolescents, scores of 0-4, 5-10, 11-14, 15-19, and ≥20 indicate absence of, mild, moderate, moderately severe, and severe depression, respectively [41].

Symptoms of diabetes-related disordered eating will be measured using the Diabetes Eating Problem Survey-Revised (DEPS-R) version [42], a diabetes-specific measure of disordered eating symptoms that is valid for use in adolescents with T1D [42]. The DEPS-R is scored from 0-80, with higher scores indicating more disordered eating behaviors, and a score of ≥20 is suggestive of disordered eating behaviors [42]. The use of a diabetes-specific tool to measure disordered eating is important, as generic measures may overestimate the prevalence of disordered eating symptoms given that diabetes management involves an emphasis on food intake and carbohydrate counting and that insulin restriction is not captured in generic measures [43].

Suicidal ideation will be assessed using 1 question on the PHQ-9, which asks the respondent if they have had thoughts that they would be better off dead or of hurting themselves in the previous 2 weeks. This question is scored on a scale from 0 (not at all) to 3 (nearly every day). In line with previous use of the PHQ-9 [44], we will consider any score of >0 to be indicative that suicidal ideation is present.

Our exploratory outcomes are the effects of time, HbA_1c_, time in range, and effect of gender. We will assess whether the effect of the MSC-T intervention on DD changes over time by exploring the interaction of time (ie, study time point) with DD. We will assess both HbA_1c_ and time in range, as not all our clinic patients wear a continuous glucose monitor (CGM), which is necessary to calculate time in range (>70% of our clinic patients wear a CGM).

HbA_1c_ is a measure of the average blood glucose levels over the previous 3 months and is a routinely followed biochemical marker used to gauge metabolic control in individuals with T1D. We will explore changes in HbA_1c_ over the duration of our trial, as mental health morbidity has been associated with higher HbA_1c_ levels [45,46]. Time in range is a metric that denotes the proportion of time that a person’s glucose level is within a desired target range, calculated using a CGM. A total of 14 days of data from CGM provides a good approximation of glucose data [47]; time in range will allow us to evaluate the glucose control at the various time points. We will collect CGM data at all study visits. Given the web-based nature of the trial combined with the high proportion of our clinic population that uses CGM, which is a more reliable measure of metabolic control [48], we will minimize additional blood collection by recording results from clinically collected HbA_1c_ at baseline and 1 year.

### Safety Parameters and Description of Risk

All the mental health outcomes being assessed are self-reported by participants directly into the study database. The principal investigator or delegate and study coordinator will be immediately alerted through an automated REDCap algorithm when (1) an individual self-reports as having suicidal ideation >0 on question 9 of the PHQ-9 or (2) an individual intentionally does not fill out the suicidality question on the PHQ-9. The principal investigator, delegate, or study coordinator will contact the participant’s endocrinologist or the endocrinologist on call, who will conduct a same-day risk assessment using the Ask Suicide-Screening Questionnaire (ASQ) [49]. If the participant is judged to have acute suicidality or suicidal ideation as per the ASQ, the assessor will either suggest a referral to the emergency department or contact the psychiatrist on call, who will suggest clinical follow-up as appropriate. The assessor will report the results of the ASQ screening back to the study coordinator within 1 day. Participants with acute suicidality as per the ASQ will then be excluded or withdrawn from the study.

### Trial Management

To foster an environment where the participants feel comfortable, the web-based MSC-T intervention will be conducted with groups of 12-18 participants of the same age group [18]. To ensure that there is no exclusion of youth based on socioeconomic status or access to technology, participants who do not have a computer, laptop, tablet, or webcam to attend the web-based sessions will be loaned a study tablet, and mobile internet devices will be provided to those who do not have high-speed internet at home. All participants will receive a subsidy to offset the cost of increased home internet use for the duration of the study. To maximize participant engagement, the trial coordinator will provide weekly reminders for the duration of the program through SMS text messages or email, depending on the participant’s preference.

At each follow-up visit (Table 3), the study coordinator will review the interim medical history using a study case report form and have the participants complete the study questionnaires.

**Table 3:** Schedule of activities.

Procedures	Baseline	8 weeks (±2 weeks)	3 months (±2 weeks)	6 months (±4 weeks)	12 months (±4 weeks)
Informed consent	✓				
Baseline demographics questionnaire	✓				
Problem Areas in Diabetes-Teen version (diabetes distress)	✓	✓	✓	✓	✓
Self-compassion Scale for Youth (self-compassion)	✓	✓	✓	✓	✓
Intervention satisfaction survey^a^		✓			
Patient Health Questionnaire (depression and suicidality)	✓	✓	✓	✓	✓
Generalized Anxiety 7-item scale (anxiety)	✓	✓	✓	✓	✓
Diabetes Eating Problem Survey-Revised version (eating disorders)	✓	✓	✓	✓	✓
Hemoglobin A_1C_	✓				✓
Time in range	✓	✓	✓	✓	✓
Review interval medical history	✓	✓	✓	✓	✓

^a^The intervention satisfaction survey will be given to the participants in the mindful self-compassion for teens (MSC-T) intervention group only.

### Engagement of People With Lived Experience of T1D

To ensure that this study is feasible, interesting, and relevant to youth with T1D, we have engaged and plan to continue to engage with people with lived experience throughout all stages of the study. We have 2 people with lived experience as collaborators (SH and OI), both of whom are former patients of the CHEO diabetes clinic, who will help spearhead engagement strategies. Multimedia Appendix 1 provides more information about our people with lived experience engagement activities.

### Equity, Diversity, and Inclusion Considerations

We have considered gender and diversity in all aspects of this study, from recruitment through to knowledge translation. Multimedia Appendix 2 [4-6,50-52] provides more information about our equity, diversity, and inclusion considerations.

### Statistical Plan

#### Sample Size

Our target sample size is 70 participants per group (140 participants total). This sample size achieves 80% power to detect group differences of 12 points or more [53] using a 2-sided significance level of 5% [26]. In this calculation, we assumed a mean PAID-T score of 73 in the waitlist control group with a SD of 26 [54]. A 30% attrition rate, based on previous relevant trials [55], was also considered.

#### Statistical Analysis

For the MSC-T and waitlist control groups, the following will be summarized using descriptive statistics: participant baseline characteristics, mental health outcome measures, and self-compassion score responses from each study time point, and for the MSC-T group, we will summarize the results of the intervention satisfaction survey and session attendance.

Our main analyses will be performed based on the intention-to-treat principle. We will also complete per-protocol analyses where we will exclude participants based on nonadherence to the intervention (defined as attending fewer than 6 out of 8 MSC-T sessions, in line with previous work done using MSC-T [56]).

#### Primary Analysis for the Primary Outcome

The primary outcome will be analyzed using a linear regression model adjusted for age and baseline DD to assess the effect of the MSC-T intervention on DD at the 3-month time point. We will adjust for age to account for the variability in the developmental stage of each participant, which may impact their response to the mindful self-compassion (MSC) program. We will also account for baseline levels of DD as we are assessing the change in DD from baseline to 3 months.

#### Secondary Bayesian Analysis for the Primary Outcome

Although our trial is powered to detect a moderate effect size of 12 points on the PAID-T, we acknowledge that a smaller effect size is still clinically meaningful. Bayesian methods focus on providing plausible values for the intervention effect that are compatible with both the observed data and previous knowledge and beliefs. Bayesian analysis enables the use of both noninformative priors that minimize the influence of priors on the statistical inference and informative priors that are guided by existing evidence or a range of collective expertise from investigators regarding the belief or skepticism of the intervention’s efficacy. Thus, a secondary Bayesian analysis will complement our frequentist analysis in the interpretation of our randomized trial results [57]. Following existing Bayesian analysis reporting guidelines [58], we will conduct a prespecified Bayesian linear regression analysis to examine the probability that the MSC-T intervention reduces DD in a clinically meaningful way compared with the control group based on the trial results.

The main objective of the Bayesian analysis is to determine the posterior probability (also known as an updated probabilistic belief) that the MSC-T intervention (1) affects PAID-T scores and (2) reduces DD by between-group differences on the PAID-T of 7-15 points (eg, effect size of 0.27-0.5), given the prior distributions of the treatment effect and the observed data from the trial. We considered an effect size of 0.27 as the minimum threshold for potential clinical relevance, given the strong relationship between DD and clinical outcomes [3], and based on the expert opinions of our team of experienced diabetologists.

To reflect our uncertainty around the effect of the MSC-T intervention, we will use a range of priors representing varying degrees of enthusiasm and skepticism (Multimedia Appendix 3). In the Bayesian regression analysis, noninformative priors will be used for nuisance parameters, including the regression intercept terms and the parameters that do not quantify the MSC intervention effectiveness. The convergence of Bayesian estimation will be examined using trace plots and the R^ convergence index with a cut-off of 1.01 [59]. With the trial data, we will estimate the 95% credible intervals using Markov chain Monte Carlo (MCMC) sampling techniques with at least 2 parallel chains [58] and report the estimated posterior probabilities for different magnitudes of treatment effects as specified in Multimedia Appendix 3. The analysis will be conducted in R statistical software [24], and Bayesian analysis will be performed using Stan MCMC (Stan Development Team) [60].

#### Analyses for Secondary Outcomes

Linear regression models and generalized linear regression models (for binary or categorical secondary outcomes), adjusting the same covariates as the primary analysis, will be used to assess the effect of the MSC-T intervention on our secondary outcomes of (1) depression, anxiety, disordered eating symptoms, and suicidal ideation at 3 months; and (2) DD, depression, anxiety, disordered eating symptoms, and suicidal ideation at 12 months.

#### Analyses for Exploratory Outcomes

To explore the effect of the MSC-T program on DD over time, we will use the 3-, 6-, and 12-month data to fit a linear mixed effects model, adjusted for time, age, baseline DD, an interaction term for time and group, and a random intercept (to account for repeated measures).

Exploratory outcome HbA_1c_ at 12 months will be modeled in a linear regression adjusted for age and baseline HbA_1c_. Linear mixed effects models, adjusted for age, baseline time in range, and a random intercept, will be used to assess the effect of the MSC-T intervention on the repeated measure time in range. Interaction terms between group and gender will be included in a separate model to investigate how the MSC-T intervention differs based on self-reported gender (collapsed to male, female, and gender-diverse for the purposes of analysis) at the primary end point.

#### Sensitivity Analysis on Missing Data

Differences in characteristics between participants with no recorded outcome measurements and those with at least 1 recorded follow-up measurement will be assessed. For participants with at least 1 available data point, we will input missing outcome data following best-case and worst-case scenarios, where best-case is defined as successfully achieving clinically meaningful improvement in PAID-T scores and worst-case is defined as failure to achieve clinically meaningful improvement in PAID-T scores.

## Results

Study recruitment began in October 2022 and was completed in March 2023, with 141 participants enrolling in the trial. Data collection will be ongoing until March 2024. The first results are expected in June 2024.

## Discussion

### Expected Findings

In this study, we will evaluate MSC-T as a way to improve DD and mental health outcomes among adolescents with T1D. We will also explore the impact of the MSC-T intervention on glycemic control. In adolescents with T1D, psychological interventions have largely targeted improvement in diet and insulin regimen adherence and strategies to cope with specific stressors [61]. While some approaches, including cognitive behavioral therapy (CBT), have shown promise for improving diabetes management, there is limited evidence that these traditional approaches translate to improved coping with distress [61]. A recent review [62] on the use of CBT for stress and coping in youth with T1D showed conflicting results, with a number of studies showing no improvement in stress [63] or DD [64]. CBT may also highlight potential causes of distress related to diabetes that the teenager has not yet considered without providing adequate skills and practices to manage these emotions [64].

We hypothesize that the combination of mindfulness and self-compassion practices in the MSC-T program will result in improved DD and mental health outcomes for adolescents enrolled in the program. We also anticipate that these improvements may translate to improved clinical outcomes through glycemic control.

### Strengths and Limitations

To our knowledge, this is the first randomized controlled trial using the MSC-T intervention to address DD in adolescents with T1D. One of our instructors (KB) is a person with lived experience of T1D; having an instructor who is also a person with lived experience of the condition has been identified as a key component in previous studies using MSC interventions [56]. The longer-term follow-up of 12 months is also a clear strength. Throughout adolescence, youth are typically required to become more independent with their diabetes self-management while receiving diminished support from their caregivers. Mental health morbidity, including DD, is associated with poor glycemic control, diabetic ketoacidosis, and hypoglycemia in youth with T1D [7,65,66]. The MSC-T program will help adolescents with T1D develop skills to better cope with their emotions around managing their diabetes.

There are, however, some potential limitations to this study. The trial is single-blind; participants and the trial coordinator are not blinded to their assignment group. However, the statistician conducting the primary analysis will be blinded to participant allocation to the MSC-T intervention or waitlist control group. Given that the intervention is web-based, it does require the use of high-speed internet and a device that has webcam functionality, which may make it difficult for some youth to attend should these items be lacking. However, in Canada and the United States, over 96% [67] and 95% [68] of youth and emerging adults own smartphones, respectively, suggesting that if the program were to be scaled nationally or internationally, attending such a program would be feasible for the majority of North Americans.

### Conclusions

Our proposed research has the potential to lower DD among youth with T1D by increasing their mindfulness-based self-compassion. We expect that our work will enhance the psychological well-being of youth with T1D, providing them with self-compassion skills to face adversity as they develop into young adults, ultimately leading them to live happier and healthier lives. If effective, further research should be done toward the implementation of MSC-T into standard clinical care for youth with T1D through partnerships with diabetes organizations.

## Appendix

Multimedia Appendix 1 Plan for engagement of people with lived experience (PWLE) of type 1 diabetes.

Multimedia Appendix 2 Equity, diversity, and inclusion (EDI) considerations.

Multimedia Appendix 3 Characteristics of reference prior probability distributions representing prior beliefs about primary outcome effect size.

## Abbreviations

ASQ: Ask Suicide-Screening Questionnaire
CBT: cognitive behavioral therapy
CGM: continuous glucose monitor
CHEO: Children’s Hospital of Eastern Ontario
DD: diabetes distress
DEPS-R: Diabetes Eating Problem Survey-Revised
DSM-IV: Diagnostic and Statistical Manual of Mental Disorders, Fourth Edition
GAD-7: Generalized Anxiety Disorder 7-item
HbA1c: hemoglobin A1c
MCMC: Markov chain Monte Carlo
MSC: mindful self-compassion
MSC-T: mindful self-compassion for teens
PAID-T: Problem Areas in Diabetes-Teen
PHQ-9: Patient Health Questionnaire-9
REDCap: Research Electronic Data Capture
T1D: type 1 diabetes

## References

[1] Kessler RC, Amminger GP, Aguilar-Gaxiola S, Alonso J, Lee S, Ustün TB (2007). Age of onset of mental disorders: a review of recent literature. Curr Opin Psychiatry.

[2] Polonsky WH, Anderson BJ, Lohrer PA, Welch G, Jacobson AM, Aponte JE, Schwartz CE (1995). Assessment of diabetes-related distress. Diabetes Care.

[3] Snoek FJ, Bremmer MA, Hermanns N (2015). Constructs of depression and distress in diabetes: time for an appraisal. Lancet Diabetes Endocrinol.

[4] Lašaitė L, Dobrovolskienė R, Danytė E, Stankutė I, Ražanskaitė-Virbickienė D, Schwitzgebel V, Marčiulionytė D, Verkauskienė R (2016). Diabetes distress in males and females with type 1 diabetes in adolescence and emerging adulthood. J Diabetes Complications.

[5] Cechetti JV, Puñales M, da Cunha LZV, Rigo L (2020). Emotional distress in patients with type 1 diabetes mellitus. Spec Care Dentist.

[6] Forsander G, Bøgelund M, Haas J, Samuelsson U (2017). Adolescent life with diabetes-gender matters for level of distress. Experiences from the national TODS study. Pediatr Diabetes.

[7] Hagger V, Hendrieckx C, Sturt J, Skinner TC, Speight J (2016). Diabetes distress among adolescents with type 1 diabetes: a systematic review. Curr Diab Rep.

[8] Davidson M, Penney ED, Muller B, Grey M (2004). Stressors and self-care challenges faced by adolescents living with type 1 diabetes. Appl Nurs Res.

[9] Pallayova M, Taheri S (2014). Targeting diabetes distress: the missing piece of the successful type 1 diabetes management puzzle. Diabetes Spectr.

[10] Neff K (2003). Self-compassion: an alternative conceptualization of a healthy attitude toward oneself. Self Identity.

[11] Muris P, Meesters C, Pierik A, de Kock B (2016). Good for the self: self-compassion and other self-related constructs in relation to symptoms of anxiety and depression in non-clinical youths. J Child Fam Stud.

[12] Prentice K, Rees C, Finlay-Jones A (2021). Self-compassion, wellbeing, and distress in adolescents and young adults with chronic medical conditions: the mediating role of emotion regulation difficulties. Mindfulness (N Y).

[13] Ferrari M, Dal Cin M, Steele M (2017). Self-compassion is associated with optimum self-care behaviour, medical outcomes and psychological well-being in a cross-sectional sample of adults with diabetes. Diabet Med.

[14] Friis AM, Johnson MH, Cutfield RG, Consedine NS (2016). Kindness matters: a randomized controlled trial of a mindful self-compassion intervention improves depression, distress, and HbA1c among patients with diabetes. Diabetes Care.

[15] MacBeth A, Gumley A (2012). Exploring compassion: a meta-analysis of the association between self-compassion and psychopathology. Clin Psychol Rev.

[16] Zessin U, Dickhäuser O, Garbade S (2015). The relationship between self-compassion and well-being: a meta-analysis. Appl Psychol Health Well Being.

[17] Neff KD, Germer CK (2013). A pilot study and randomized controlled trial of the mindful self-compassion program. J Clin Psychol.

[18] Campo RA, Bluth K, Santacroce SJ, Knapik S, Tan J, Gold S, Philips K, Gaylord S, Asher GN (2017). A mindful self-compassion videoconference intervention for nationally recruited posttreatment young adult cancer survivors: feasibility, acceptability, and psychosocial outcomes. Support Care Cancer.

[19] Ni Y, Ma L, Li J (2020). Effects of mindfulness-based stress reduction and mindfulness-based cognitive therapy in people with diabetes: a systematic review and meta-analysis. J Nurs Scholarsh.

[20] Grylli V, Wagner G, Hafferl-Gattermayer A, Schober E, Karwautz A (2005). Disturbed eating attitudes, coping styles, and subjective quality of life in adolescents with Type 1 diabetes. J Psychosom Res.

[21] Harris PA, Taylor R, Minor BL, Elliott V, Fernandez M, O'Neal L, McLeod L, Delacqua G, Delacqua F, Kirby J, Duda SN (2019). The REDCap consortium: building an international community of software platform partners. J Biomed Inform.

[22] Harris PA, Taylor R, Thielke R, Payne J, Gonzalez N, Conde JG (2009). Research Electronic Data Capture (REDCap)--a metadata-driven methodology and workflow process for providing translational research informatics support. J Biomed Inform.

[23] Neff KD, Bluth K, Tóth-Király I, Davidson O, Knox MC, Williamson Z, Costigan A (2021). Development and validation of the self-compassion scale for youth. J Pers Assess.

[24] (2023). The R project for statistical computing. The R Foundation.

[25] Sanders RA (2013). Adolescent psychosocial, social, and cognitive development. Pediatr Rev.

[26] Hulley SB, Cummings SR, Browner WS, Grady DG, Newman TB (2013). Designing Clinical Research. 4th Edition.

[27] Pampalon R, Hamel D, Gamache P, Philibert MD, Raymond G, Simpson A (2012). An area-based material and social deprivation index for public health in Québec and Canada. Can J Public Health.

[28] Pampalon R, Hamel D, Gamache P, Raymond G (2009). A deprivation index for health planning in Canada. Chronic Dis Can.

[29] Fox KR, Choukas-Bradley S, Salk RH, Marshal MP, Thoma BC (2020). Mental health among sexual and gender minority adolescents: examining interactions with race and ethnicity. J Consult Clin Psychol.

[30] Salk RH, Thoma BC, Choukas-Bradley S (2020). The gender minority youth study: overview of methods and social media recruitment of a nationwide sample of U.S. cisgender and transgender adolescents. Arch Sex Behav.

[31] (2022). Guidance on the use of standards for race-based and indigenous identity data collection and health reporting in Canada. Canadian Institute for Health Information.

[32] Weissberg-Benchell J, Antisdel-Lomaglio J (2011). Diabetes-specific emotional distress among adolescents: feasibility, reliability, and validity of the problem areas in diabetes-teen version. Pediatr Diabetes.

[33] Hagger V, Hendrieckx C, Cameron F, Pouwer F, Skinner TC, Speight J (2017). Cut points for identifying clinically significant diabetes distress in adolescents with type 1 diabetes using the PAID-T: results from diabetes MILES youth-Australia. Diabetes Care.

[34] Shapiro JB, Vesco AT, Weil LEG, Evans MA, Hood KK, Weissberg-Benchell J (2018). Psychometric properties of the problem areas in diabetes: teen and parent of teen versions. J Pediatr Psychol.

[35] Weissberg-Benchell J, Vesco AT, Rychlik K (2019). Diabetes camp still matters: relationships with diabetes-specific distress, strengths, and self-care skills. Pediatr Diabetes.

[36] Hood KK, Iturralde E, Rausch J, Weissberg-Benchell J (2018). Preventing diabetes distress in adolescents with type 1 diabetes: results 1 year after participation in the STePS Program. Diabetes Care.

[37] Spitzer RL, Kroenke K, Williams JBW, Löwe B (2006). A brief measure for assessing generalized anxiety disorder: the GAD-7. Arch Intern Med.

[38] Mossman SA, Luft MJ, Schroeder HK, Varney ST, Fleck DE, Barzman DH, Gilman R, DelBello MP, Strawn JR (2017). The Generalized Anxiety Disorder 7-item scale in adolescents with generalized anxiety disorder: Signal detection and validation. Ann Clin Psychiatry.

[39] Nguyen LA, Pouwer F, Winterdijk P, Hartman E, Nuboer R, Sas T, de Kruijff I, Bakker-Van Waarde W, Aanstoot HJ, Nefs G (2021). Prevalence and course of mood and anxiety disorders, and correlates of symptom severity in adolescents with type 1 diabetes: results from diabetes LEAP. Pediatr Diabetes.

[40] Watson SE, Spurling SE, Fieldhouse AM, Montgomery VL, Wintergerst KA (2020). Depression and anxiety screening in adolescents with diabetes. Clin Pediatr (Phila).

[41] Richardson LP, McCauley E, Grossman DC, McCarty CA, Richards J, Russo JE, Rockhill C, Katon W (2010). Evaluation of the patient health questionnaire-9 item for detecting major depression among adolescents. Pediatrics.

[42] Markowitz JT, Butler DA, Volkening LK, Antisdel JE, Anderson BJ, Laffel LMB (2010). Brief screening tool for disordered eating in diabetes: internal consistency and external validity in a contemporary sample of pediatric patients with type 1 diabetes. Diabetes Care.

[43] Wisting L, Frøisland DH, Skrivarhaug T, Dahl-Jørgensen K, Rø O (2013). Psychometric properties, norms, and factor structure of the diabetes eating problem survey-revised in a large sample of children and adolescents with type 1 diabetes. Diabetes Care.

[44] Majidi S, O'Donnell HK, Stanek K, Youngkin E, Gomer T, Driscoll KA (2020). Suicide risk assessment in youth and young adults with type 1 diabetes. Diabetes Care.

[45] Rechenberg K, Whittemore R, Grey M (2017). Anxiety in youth with type 1 diabetes. J Pediatr Nurs.

[46] Moore SM, Hackworth NJ, Hamilton VE, Northam EP, Cameron FJ (2013). Adolescents with type 1 diabetes: parental perceptions of child health and family functioning and their relationship to adolescent metabolic control. Health Qual Life Outcomes.

[47] Riddlesworth TD, Beck RW, Gal RL, Connor CG, Bergenstal RM, Lee S, Willi SM (2018). Optimal sampling duration for continuous glucose monitoring to determine long-term glycemic control. Diabetes Technol Ther.

[48] Beck RW, Connor CG, Mullen DM, Wesley DM, Bergenstal RM (2017). The fallacy of average: how using HbA alone to assess glycemic control can be misleading. Diabetes Care.

[49] Horowitz LM, Bridge JA, Teach SJ, Ballard E, Klima J, Rosenstein DL, Wharff EA, Ginnis K, Cannon E, Joshi P, Pao M (2012). Ask Suicide-Screening Questions (ASQ): a brief instrument for the pediatric emergency department. Arch Pediatr Adolesc Med.

[50] Fegan-Bohm K, Minard CG, Anderson BJ, Butler AM, Titus C, Weissberg-Benchell J, Hilliard ME (2020). Diabetes distress and HbA1c in racially/ethnically and socioeconomically diverse youth with type 1 diabetes. Pediatr Diabetes.

[51] Robinson ME, Simard M, Larocque I, Shah J, Nakhla M, Rahme E (2020). Risk of psychiatric disorders and suicide attempts in emerging adults with diabetes. Diabetes Care.

[52] Brookes ST, Whitely E, Egger M, Smith GD, Mulheran PA, Peters TJ (2004). Subgroup analyses in randomized trials: risks of subgroup-specific analyses; power and sample size for the interaction test. J Clin Epidemiol.

[53] Zoffmann V, Vistisen D, Due-Christensen M (2015). Flexible guided self-determination intervention for younger adults with poorly controlled type 1 diabetes, decreased HbA1c and psychosocial distress in women but not in men: a real-life RCT. Diabet Med.

[54] Iturralde E, Rausch JR, Weissberg-Benchell J, Hood KK (2019). Diabetes-related emotional distress over time. Pediatrics.

[55] Karlson CW, Rapoff MA (2009). Attrition in randomized controlled trials for pediatric chronic conditions. J Pediatr Psychol.

[56] Bluth K, Lathren C, Clepper-Faith M, Larson LM, Ogunbamowo DO, Pflum S (2021). Improving mental health among transgender adolescents: implementing mindful self-compassion for teens. J Adolesc Res.

[57] Lewis RJ, Angus DC (2018). Time for clinicians to embrace their inner bayesian?: Reanalysis of results of a clinical trial of extracorporeal membrane oxygenation. JAMA.

[58] van Doorn J, van den Bergh D, Böhm U, Dablander F, Derks K, Draws T, Etz A, Evans NJ, Gronau QF, Haaf JM, Hinne M, Kucharský Š, Ly A, Marsman M, Matzke D, Gupta ARKN, Sarafoglou A, Stefan A, Voelkel JG, Wagenmakers EJ (2021). The JASP guidelines for conducting and reporting a bayesian analysis. Psychon Bull Rev.

[59] Vehtari A, Gelman A, Simpson D, Carpenter B, Bürkner PC (2021). Rank-normalization, folding, and localization: an improved Rˆ for assessing convergence of MCMC (with discussion). Bayesian Anal.

[60] (2023). Stan user’s guide version 2.33. Stan Development Team.

[61] Aljawarneh YM, Al-Qaissi NM, Ghunaim HY (2020). Psychological interventions for adherence, metabolic control, and coping with stress in adolescents with type 1 diabetes: a systematic review. World J Pediatr.

[62] Rechenberg K, Koerner R (2021). Cognitive behavioral therapy in adolescents with type 1 diabetes: an integrative review. J Pediatr Nurs.

[63] Serlachius AS, Scratch SE, Northam EA, Frydenberg E, Lee KJ, Cameron FJ (2016). A randomized controlled trial of cognitive behaviour therapy to improve glycaemic control and psychosocial wellbeing in adolescents with type 1 diabetes. J Health Psychol.

[64] Salamon KS, Hains AA, Fleischman KM, Davies WH, Kichler J (2010). Improving adherence in social situations for adolescents with type 1 diabetes mellitus (T1DM): a pilot study. Prim Care Diabetes.

[65] Plener PL, Molz E, Berger G, Schober E, Mönkemöller K, Denzer C, Goldbeck L, Holl RW (2015). Depression, metabolic control, and antidepressant medication in young patients with type 1 diabetes. Pediatr Diabetes.

[66] Law GU, Walsh J, Queralt V, Nouwen A (2013). Adolescent and parent diabetes distress in type 1 diabetes: the role of self-efficacy, perceived consequences, family responsibility and adolescent-parent discrepancies. J Psychosom Res.

[67] (2021). Smartphone personal use and selected smartphone habits by gender and age group. Statistics Canada.

[68] Anderson M, Perrin A, Jiang J, Kumar M (2019). 10% of Americans don't use the internet. Who are they?. Pew Research Center.

