# Supplementary material for: Teaching Adolescents With Type 1 Diabetes Self-Compassion (TADS) to Reduce Diabetes Distress: Protocol for a Randomized Controlled Trial
Source: JMIR Res Protoc. 2023 Dec 26;12:e53935. doi: 10.2196/53935 (PMC10777281; doi:10.2196/53935)
Supplement: Multimedia Appendix 1 [file resprot_v12i1e53935_app1.docx]

# Appendix A: Plan for engagement of people with lived experience (PWLE) of type 1 diabetes

To ensure that our study is feasible, interesting, and relevant to youth with type 1 diabetes (T1D), we have engaged, and plan to continue to engage with PWLE throughout all stages of the proposed study. We have two PWLE collaborators, both of whom are former patients of the Children’s Hospital of Eastern Ontario (CHEO) diabetes clinic, who will help spearhead engagement strategies.

Study design: We consulted with both PWLE collaborators about relevant aspects of the study design, such as the design of the planned intervention (delivery method, number and duration of sessions) and the feasibility of getting adolescents to participate. They also reviewed the protocol through their lens as PWLEs. In addition, we consulted with the CHEO Patient Family Advisory Committee concerning elements of our study design and Equity, Diversity and Inclusion (EDI) strategies to ensure we were being as inclusive as possible in our recruitment, intervention design, and knowledge dissemination strategies.

Trial Oversight: To provide guidance and oversight throughout the trial, we will establish a PWLE Oversight Committee (co-chaired by one PWLE collaborator and the study principal investigator). The committee will meet quarterly to review the trial progress and provide input regarding elements of the study that are going well, elements that could be improved, and how these might be improved. Meetings will be informal and casual (there will be no formal agenda), with the aim of fostering a comfortable environment where individuals with T1D can connect while also providing meaningful input regarding the operationalization and progress of the study. Members of the oversight committee will also be invited to attend the investigator meetings and given the opportunity to share their story and events that led them to want to be involved in clinical research.
