# Supplementary material for: Teaching Adolescents With Type 1 Diabetes Self-Compassion (TADS) to Reduce Diabetes Distress: Protocol for a Randomized Controlled Trial
Source: JMIR Res Protoc. 2023 Dec 26;12:e53935. doi: 10.2196/53935 (PMC10777281; doi:10.2196/53935)
Supplement: Multimedia Appendix 2 [file resprot_v12i1e53935_app2.docx]

# Appendix B: Equity, Diversity & Inclusion (EDI) Considerations

We have considered gender and diversity in all aspects of our study, from recruitment through to knowledge translation.

Recruitment: Given the Children’s Hospital of Eastern Ontario (CHEO)’s large catchment area, the CHEO diabetes clinic already sees youth of all backgrounds. Our study involves a virtual program using Zoom Healthcare (Zoom Video Communications Inc., San Jose, California, USA). In order to ensure that ability to attend the sessions is not a barrier to participation in the study, we will purchase study tablets to lend to participants who do not have access to a computer/laptop/tablet at home. We will also provide a stipend to offset the increased use of the home internet that will result from participation in the study and provide mobile internet devices to those who do not have high-speed internet at home. These considerations will provide equitable access to the study intervention in both groups of the study for any individual who wish to participate.

Study procedures: We will have all study questionnaires available in both English and French, keeping in line with the bilingual nature of the programs and care offered at CHEO.

Data analysis: Previous studies have shown that socioeconomic status, race/ethnicity, gender identity, and rurality are associated with poorer psychiatric outcomes for individuals with T1D [50,51]. We plan to conduct formal analysis to determine if the effect of the MSC intervention differs based on gender (female, male, gender-diverse people), since adolescent females with T1D are known to experience greater diabetes distress compared to males [4-6]. However, it is unknown whether the response to the intervention will differ by gender. Therefore, we will conduct a linear regression model with gender as an interaction term, to determine if there is a significant interaction between our outcomes and gender (in other words, we are investigating if gender is an effect modifier of the MSC program). If the interaction term is significant, that suggests that gender would be an effect modifier for response to the intervention.

In order to determine if our study sample will be large enough to conduct this analysis, we had performed a simulation-based power calculation, since typical formulas to determine sample size and statistical power do not feature calculation via direct specification of the interaction effect on the mean of the continuous outcome while accounting for multiple continuous and categorical covariates. We generated the simulation following the methodology described by Brookes and colleagues [52]. We generated the outcome model to test for the interaction by including an interaction term between group and gender. This model is adjusted for age, as well as baseline diabetes distress.

We calculated simulated power using the proportion of interactions that returned significant F-test results on the interaction term. For a total sample size of 94 (47 in the wait-list control group and 47 in the MSC intervention group), we can successfully detect an interaction effect that is about 2 times the overall treatment effect at 15*2 = 30 points on diabetes distress with 80% power (Figure 2 below). Therefore, we have the required sample size to detect if gender modifies the effect of the intervention by a factor of approximately 2.

Figure 2. Size of gender interaction effect on the intervention, for a sample size of 47 in each group, at 80% power


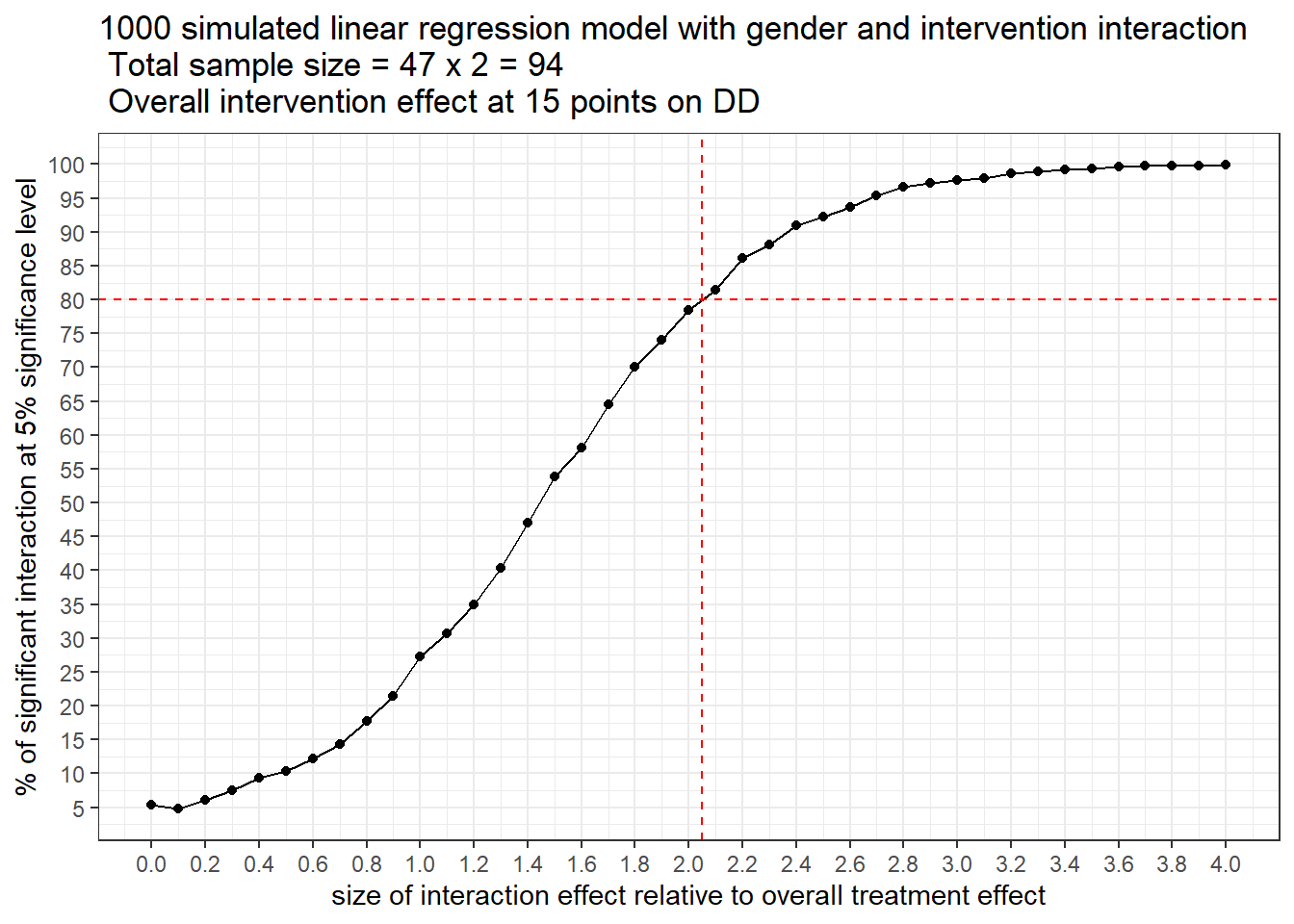


To collect the data to perform these analyses, we will provide study participants with a confidential case report form where they can optionally self-identify race/ethnicity, Indigeneity (using reporting options in line with accepted standards, see Table 4), gender identity (using gender reporting options in line with accepted standards, see Table 3). Disclosure of this information will be optional, and will not affect randomization or treatment in the study. Participants will be clearly informed of the intended use of the information collected. In order to protect the participants, all information disclosed will be strictly confidential between them and the study team; the participant can choose whether or not to share this information with their parents/guardians.
