# Supplementary material for: Teaching Adolescents With Type 1 Diabetes Self-Compassion (TADS) to Reduce Diabetes Distress: Protocol for a Randomized Controlled Trial
Source: JMIR Res Protoc. 2023 Dec 26;12:e53935. doi: 10.2196/53935 (PMC10777281; doi:10.2196/53935)
Supplement: Multimedia Appendix 3 [file resprot_v12i1e53935_app3.docx]

**Appendix C**

Table 1. Characteristics of reference prior probability distributions representing prior beliefs about primary outcome effect size. The range of values was selected to represent a) the minimum difference that our group of expert diabetologists agreed would be a clinically meaningful change on the PAID-T, and b) published data of the effect size of treatment effects in adults.

| **Prior belief** | **Assumed treatment effect** | **Assumed SD of effect** | **Probability of intervention effect less than the specified threshold of the between group difference on the PAID-T (Δ=difference; d=effect size)** | | | | | | **Rationale for specifying distribution characteristics** |
| --- | --- | --- | --- | --- | --- | --- | --- | --- | --- |
|  |  |  | **Δ <-15**  **(*d=0.58)*** | **Δ < -12**  **(*d=0.46)*** | **Δ < -10.4**  **(*d=0.4)*** | **Δ < -7.8**  **(*d=0.3)*** | **Δ < -7.0**  **(*d=0.27)*** | **Δ < 0**  **(d=0)** |  |
| Non-informative | 0 | 100 | 44% | 45% | 46% | 47% | 47% | 50% | All possible values for treatment effect are equally likely |
| Enthusiastic | -12 | 10 | 38% | 50% | 56% | 66% | 69% | 88% | Based on published data sources, the treatment effect of the intervention on the outcome measure is approximately -12 with a derived standard deviation of 10 |
| Skeptical | 0 | 9.1 | 5% | 9% | 13% | 20% | 22% | 50% | Probability of observing a treatment effect greater than that assumed in the trial design (effect size = 0.46) is 5%; the probability of any benefit or harm is equal |
